# Redifferentiating Thyroid Cancer: Selumetinib-enhanced Radioiodine Uptake in Thyroid Cancer

**DOI:** 10.4274/2017.26.suppl.09

**Published:** 2017-01-09

**Authors:** Steven M. Larson, Joseph R. Osborne, Ravinder K. Grewal, R. Michael Tuttle

**Affiliations:** 1 Memorial Sloan Kettering Cancer Center, New York, USA

**Keywords:** Redifferentiation, thyroid cancer, selumetinib, radioiodine

## Abstract

In a recent article, we reported a restorative therapeutic intervention that turned individual thyroid cancer lesions into more efficient tissues for taking up radioactive iodine (RAI), resulting in clinically significant and durable responses. A group of Iodine-131 refractory thyroid cancer patients were treated with the MEK tyrosine kinase inhibitor (TKI) selumetinib, and RAI uptake was restored in a subset of patients. We employed Iodine-124 positron emission tomography to measure radiation absorbed dose, on a lesion by lesion basis. The process can be thought of as a re-differentiation of the cancer toward a more nearly normal state most like the tissue from which the cancer arose. Remarkably, in its own way, a change was detected within a few weeks of treatment, restoring uptake with therapeutically effective levels of RAI and in some patients, previously completely refractory to radioiodine treatment. In this article, we summarize the basic work that led to this seminal study, and make the case for lesional dosimetry in thyroid cancer with Iodine-124 as a new optimal radiotracer for precision medicine in patients with well differentiated thyroid cancer.

## Abstract (tr)

Yakın tarihli bir yayında bireysel tiroid kanseri lezyonlarını radyoaktif iyot (RAİ) tutulumu açısından daha etkin dokulara çeviren ve klinik olarak anlamlı ve kalıcı cevaba yol açan restoratif terapötik bir girişim bildirdik. Bir grup İyot-131 refrakter tiroid kanseri hastası MEK tirozin kinaz inhibitörü selumetinib ile tedavi edildi ve bir kısım hastada RAI tutulumu sağlandı. Lezyon-lezyona temelinde İyot-124 pozitron emisyon tomografisi ile abzorbe edilen radyasyon dozunu ölçtük. Bu işlem kanserin, ilk çıktığı dokudaki normal duruma yakın bir hale re-diferansiyasyonu olarak düşünülebilir. İlgi çekici bir şekilde birkaç hafta tedavi ile öncesinde tamamen RAİ tedavisine refrakter olan bazı hastalarda terapötik olarak efektif dozlarda RAİ tutulumu sağlandı. Bu makalede, bu çığır açıcı temel çalışma özetlenmekte ve iyi diferansiye tiroid kanser hastalarında tedavi için yeni bir optimal radyofarmasötik olarak tiroid kanserinde lezyonel dozimetri için İyot-124 kullanımı sunulmaktadır.

## INTRODUCTION

**Figure 1:**
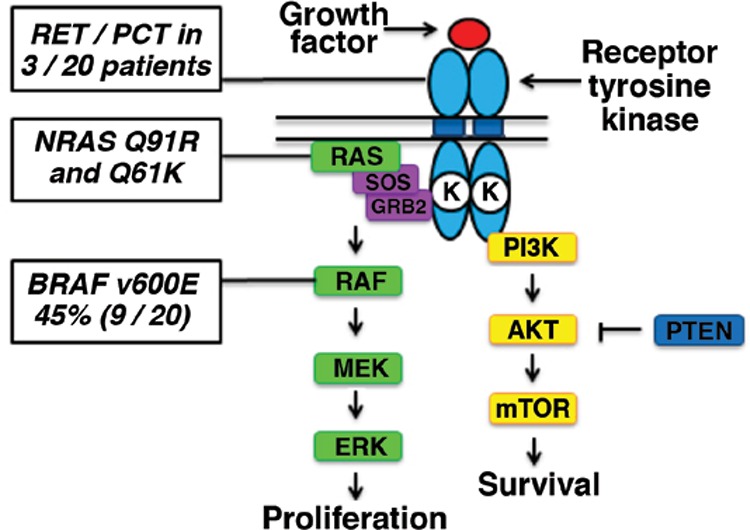
Mitogen-activated protein kinase signaling in papillary thyroid carcinoma is depicted along with statistical information regarding mutations and specifications at distinct points on the pathway. (Courtesy of James A. Fagin and Alan L. Ho, Memorial Sloan Kettering Cancer Center.)

If we think of a tissue with cancer as an unhappy family of interacting cells (in contrast to the “happy” well differentiated normal tissue), we begin to understand that the sad fact of malignant change can truly occur in many ways. Often, the culprits are diverse changes in the genetic code, which are now understood to be the basis for a significant number of common cancers in man. These cancer-causing genes, “oncogenes,” may transform cells from normal to malignant by simple alterations in base sequences that are, in turn, transformed into alterations in the protein product of the oncogene. When a mutation is the cause of the malignant transformation, this genetic change is called a “driver oncogene.” The tissue affected is said to be “oncogene-addicted” and is found when growth and even viability are dependent on the presence and continuous activity of the oncoprotein product of the specific mutation. Specific oncogenes have been identified in lung cancer, breast cancer, lymphomas, leukemias, and pediatric cancers. With regard to thyroid cancer, knowledge about the genetic lesions associated with papillary thyroid cancer has increased enormously, in the last few years. More than 95% of the driver oncogene mutations are known, and 75% of these mutations occur in the mitogen-activated protein (MAP) kinase signal transduction pathway (Figure 1).

## Integrated Genomic Characterization of Papillary Thyroid Carcinoma

Effector molecules such as MEK, which in turn signal ERK, indirectly promote cellular proliferation and growth and accompany the de-differentiation of the thyroid cell, driving it to take on the properties of neoplasia. For thyroid cancer, this means a down regulation of key mature tissue functions such as iodine uptake and the formation of thyroid hormone, as the tumor cell “goes primitive” and develops a new phenotype of sustained growth and metastasis.

## Pre-clinical Observations Setting the Stage for Re-induction Therapy

**Figure 2:**
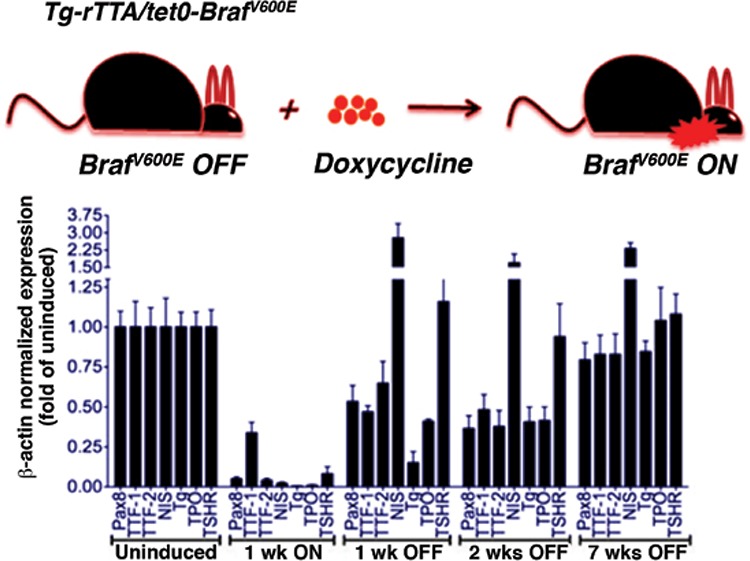
Transgenic mouse model demonstrating the effect of doxycline induction of mutant B-Raft in thyroid cells. Note that within a week of induction of the driver oncogene, the cadre of proteins responsible for uptake of radioiodine are rapidly down regulated, as the effect of the driver oncogene takes hold. If the doxycline is stopped, this effect can be ameliorated, and it is the sodium iodine symporter protein, of Na+/I- symporter, that responds most quickly (from reference 1).

The genetic mutation in the BRAF gene encodes the serine/threonine signal transduction molecule BRAF (v-raf murine sarcoma viral oncogene homolog B1), resulting in amino acid transformation at the 600 locus in the BRAF protein. This transformation occurs as the “driver mutation” in about 40% of thyroid cancers, and about 70% of melanomas. BRAF is actually an enzyme member of a signal transduction pathway, and it is this enzymatic function that transmits a message by activating through phosphorylation, the next protein in sequence, MEK [Text Box 1] (Figure 2).

My colleagues at Memorial Sloan Kettering Cancer Center developed a mouse model for thyroid cancer in order to study the effect of the BRAF driver oncogene on a molecular basis and its role in creating the tumor phenotype. The mouse model was transgenic, in which thyroid follicular cells were induced to carry a BRAF (V600E) mutation that could be activated by treatment with doxycycline. Under treatment of these special mice with doxycycline, the thyroid of the mice transformed to take on the characteristics of a poorly differentiated thyroid cancer, for example, losing the ability to take up radioactive iodine. In their seminal paper, the authors noted the following: “Strikingly, treatment with the MAPK pathway inhibitors rendered the tumor cells susceptible to a therapeutic dose of radioactive iodine. Our data show that thyroid tumors carrying *BRAF^V600E^* mutations are exquisitely dependent on the oncoprotein for viability and that genetic or pharmacological inhibition of its expression or activity is associated with tumor regression and restoration of radioactive iodine uptake in vivo in mice. These findings have potentially significant clinical ramifications” (Figure 2) (2).

Inhibitor drugs that block the activity of this “driver oncogene,” particularly in melanoma, have been developed and widely used, greatly benefiting select cases of advanced melanoma (3). This class of medications, called tyrosine kinase inhibitor drugs, or TKIs, act by blocking the enzymatic activity of a signal transduction protein. Examples include vemurafinib to block B-RAF directly and selumetinib as a blocker of the downstream MEK activity. Using this logic, the team of Chakravarty et al. (2) demonstrated proof of principle that inhibition of the MAPK pathway in the transgenic model of mutant BRAF-induced thyroid cancer partially restored radioactive iodine (RAI) uptake mechanisms within the cancer cell, sufficient to both increase RAI uptake and prolong retention in the cell. The effects were sufficient to achieve a major treatment effect on mouse thyroid cancer. This finding led to the extension of this approach to man, described in more detail below.

One feature of the preclinical paper described above was the rapid effect of the mutant BRAF gene induction on transformation of the thyroid cell into a de-differentiated state. Similarly, pharmacologic effects were also diffused and non-focal when restoring features of the differentiated state. This is consistent with direct cellular effect, which is not related to clonal selection of sensitive cells, because the maturation occurs too fast and indeed throughout the tumor mass.

There is an increasing awareness that cancers growing in the human body are capable of continuously transforming themselves, often under the pressure of specific treatments, in both fundamental and meaningful ways. This biologic transformation is especially common for patients undergoing treatment with targeted kinase inhibitor drugs (TKIs), whereby during treatment, further evolution of genetic changes may result in resistance to specific anti-tumor drugs. For example, one of the earliest targeted therapies, imatinib (trade name: Gleevec), was found to be highly effective against chronic forms of myeloid leukemia, due to specific effectiveness through binding to BCR-ABL kinase. The mode of action of imatinib was based on blocking the enzymatic kinase action of this protein. However, resistance developed in many patients through changes in the genetic makeup of the genes encoding BCR-ABL, resulting in alterations at multiple points in the protein, which in turn nullified the binding action of imatinib. The same process could take place during inhibition of mutant BRAF in thyroid cancer, by drug treatment, and the patient may ultimately escape the effects of therapy.

## Iodine-124 at Memorial Sloan Kettering Cancer Center

**Figure 3:**
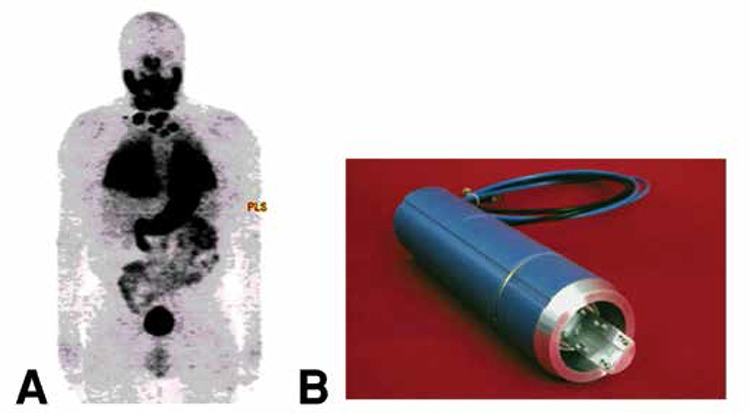
A) Reprojection positron emission tomography imaging in a patient with well differentiated thyroid cancer, metastatic to the lungs, regional neck nodes and also suspected metastases in the scapula and T-12 vertebrae. At the time of imaging, thyroglobulin levels >11,000 ng/mL; estimated dose from 250 mCi of 131I estimated at >50,000 cGy; follow-up at 13 years post-multiple 131I treatments (1500 mCi total), TG <0.2, complete clinical response. B) MSKCC (Finn) target system, used to manufacture 124I. Tellurium-124.) 15 MeV protons. 124I can be produced in significant quantities by modern hospital and radiopharmacy based cyclotrons

At MSKCC, we developed positron emission tomography (PET) imaging, as a quantitative molecular imaging tool in the study of thyroid cancer, and one of our earliest efforts was methodology for iodine-124 (^124^I) production, supported by the Department of Energy (DOE) (Figure 3) (4). We used ^124^I as a tool for characterization of targeting and dosimetry of radioantibodies, such as 3F8, an anti-GD2 antibody (5), and A33, an anti-A33 antibody (colon cancer) (6). We and others have also found this radionuclide of special benefit in defining the lesional dosimetry of iodine-131 (^131^I) therapy in man (7,8). Although ^131^I can also be imaged with cross-sectional imaging, using single-photon emission computed tomography in the post-therapy setting (9) indicates there is little doubt that ^124^I and PET imaging can detect more lesions and also quantitate more readily, especially lesions in the 1-2 cm. in diameter range, a size commonly seen in pulmonary metastases. An example of ^124^I imaging in man is shown in Figure 3, which shows a reprojection image in a patient with documented thyroid cancer metastatic to the lung, neck nodes as well as to bone.

## Re-induction of Therapeutic Levels of Radioiodine Uptake in Metastatic Thyroid Cancer in Man

**Figure 4:**
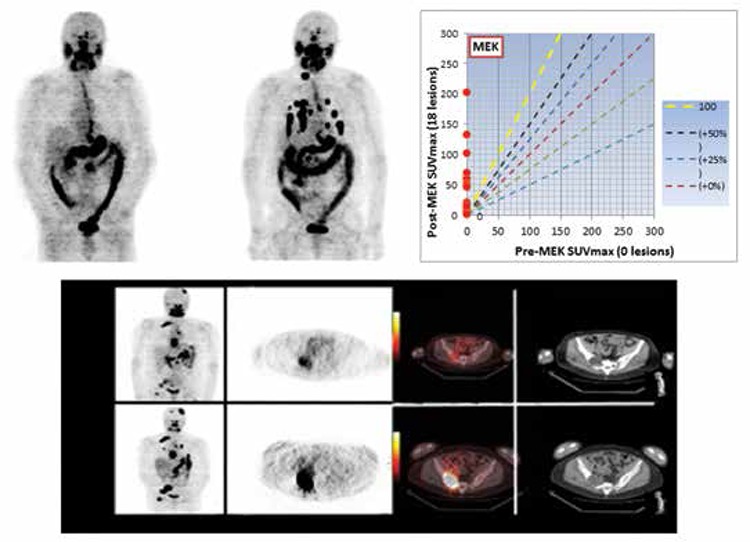
A thyroid cancer patient with widely metastatic neck nodes, pulmonary nodules, mediastinum, and osseous sites. The upper row is the baseline study before seluemetanib treatment (left), and the post four week MEK treatment image showing a number of pulmonary lesions now taking up the radioactive iodine. The graph (top, right) compares the standardized uptake values (SUV) before (abscissa) and after (ordinate) treating in quantitative terms. No lesions were detectable in terms of 124I prior to treatment and subsequently, the uptake that developed was seen. There is a different patient showing baseline (upper row) and the re-induction effects of treatment on radioiodine uptake (bottom row). There are new lesions in the lungs and the osseous lesions have greater uptake (left panel coronal image baseline, above and post-treatment below); cross-sectional imaging 124I imaged on positron emission tomography, baseline (above) and post-treatment (below). There is marked increase in the intensity of uptake in the R. pelvis, and the relationship to computed tomography findings is dramatically displayed in the fusion positron emission tomography/computed tomography, which shows a space occupying lesion in the R. ileum, now with therapeutic levels of uptake. As part of the selumetinib pilot trial, we have been able to document dose-response relationships between 124I lesional dosimetry and subsequent structural disease response as determined by Response Evaluation Criteria in Solid Tumors criteria in eight differentiated thyroid cancer patients (1).

In a 20-patient clinical pilot trial, we effectively used quantitative ^124^Iodine-NaI PET imaging to estimate individual lesion dosimetry before and after a four-week course of the serine-threonine kinase MEK inhibitor selumetinib (AZD6244) to identify patients who would benefit from additional RAI therapy and to individualize therapeutic dosing recommendations. We demonstrated that selumetinib re-induced iodine uptake in a significant subset of patients with radioiodine refractory metastatic thyroid cancer (1): 12/20 RAI-refractory patients achieved improved lesional radioiodine uptake with selumetinib therapy. Of those 12 patients, 8 had at least one tumor with ^124^I uptake that predicted delivery of ≥2,000 cGy with ≤300 mCi therapeutic ^131^I, and went on to experience tumor reductions with therapeutic ^131^I given concomitantly with selumetinib (5 Response Evaluation Criteria in Solid Tumors (RECIST) partial responses, 3 RECIST stable disease). Equally important, the lesional dosimetry also identified a subset of patients in whom high-dose RAI would not be effective, thereby avoiding unnecessary radioiodine exposure. These findings illustrated the potential role of quantitative PET imaging as a tool for documenting drug effects in vivo, and supported the concept that ^124^I PET imaging can be used to measure a threshold radiation absorbed tumor dose that will identify patients who will respond to ^131^I (Figure 4).

## The Clinical Problem of Radioactive Iodine Treatment of Thyroid Cancer: A Rationale for Lesional Dosimetry

^131^I therapy is widely used in thyroid cancer for thyroid ablation and treatment of metastatic disease. However, the standard approach of using empirical administered activities of ^131^I to treat metastatic disease without reliable measurements of lesional dosimetry leads to multiple ineffective RAI treatments in patients subsequently proven to have RAI refractory disease, as well as to sub-optimal dosing even in patients with RAI-avid disease. Since ^131^I therapy can be associated with significant toxicity (salivary gland damage, lacrimal duct obstruction, and at higher cumulative doses, myelodysplastic syndrome and leukemia), an accurate dosimetry test that predicts whether or not patients will respond to a therapeutic dose of ^131^I is sorely needed.

Distant metastases are identified in 10-15% of patients with differentiated thyroid cancer at some point in the course of their disease. For more than 50 years, RAI has been the mainstay of therapy in these patients (10). However, even in RAI-avid metastatic disease, multiple administrations of RAI are usually required to control the disease. Furthermore, RAI therapy rarely results in clinical remission except in young patients with very small volume, RAI-avid, miliary metastases. The majority of patients with structurally evident RAI-avid distant metastases will have a clinical response marked by either disease stabilization or partial regression of macroscopic disease. Then again, no prospective randomized placebo-controlled studies have established efficacy on progression-free survival or overall survival. Survival rates are less than ideal, with five-year survival rates of 80% and ten-year survival rates of 55%, even when the distant metastases are RAI-avid (11). Thus, whereas RAI may be an effective treatment in a subset of patients with “RAI-avid” distant metastases, it is seldom curative, and the tools to predict who may benefit and who may not have not been definitively established.

Compared with older series that evaluated responses to therapy on the basis of post-therapy RAI scans and chest radiographs, recent retrospective studies demonstrate that few patients with macroscopic distant metastases have clear-cut RECIST responses when the effectiveness of RAI therapy is assessed with computed tomography or magnetic resonance imaging, or with other modalities such as fludeoxyglucose-PET or the plasma biomarker thyroglobulin (12,13,14,15,16). Furthermore, it is unclear whether the “disease stabilization” seen after RAI is a treatment effect or due to the natural history of insidious growth of well differentiated. RAI-avid metastatic disease (17). It is critically important to determine if inadequate lesional dose is the reason for the relative ineffectiveness of RAI therapy. Recent indications suggest that this may indeed be the case; i.e., pre-treatment with the MEK inhibitor selumetinib prior to RAI therapy dramatically increased the lesional dose in patients with RAI-refractory metastatic thyroid cancer and resulted in a high rate of responses as determined by RECIST criteria (1). Thus, compelling evidence now indicates that inadequate lesional dose is a key limitation to the effectiveness of RAI therapy. Studies designed to quantify, enhance, and optimize lesional dose have become even more critical.

Several approaches to determining lesional dosimetry have been published (18,19) but none of them has gained acceptance into clinical practice outside of a few specialized centers. More recently, 124I-PET has emerged as a potentially clinically applicable methodology for scanning and quantifying lesional dosimetry in differentiated thyroid cancer (7,20,21). While it is clear that whole-body scanning and lesional dosimetry measurements with ^124^I are feasible, studies correlating ^124^I lesional dosimetry with the effectiveness of RAI as measured by cross-sectional imaging (which are needed to define dose-response relationships) have not been conducted.

While lesional dosimetry is a key component of optimal RAI dosing, it is also important to define the maximal tolerable dose that can be administered without complications. Optimal RAI dosing should be based on precise lesional dosimetry (to define an appropriate therapeutic dose), coupled with whole-body dosimetry studies to ensure that the proposed activity can be safely administered without unacceptable side effects. Unfortunately, in clinical practice, the selection of the RAI-administered activity is based far more on its potential toxicity than on the dose likely to be required to achieve a tumoricidal effect.

Over the last 50 years, several empiric dosing regimens that called for fixed doses of RAI at specific intervals have been shown in retrospective studies to be generally well tolerated and often effective (10,12,22). However, recent studies suggest that these empirical dosing recommendations may exceed the maximum safe radiation dose in a significant proportion of older patients with RAI-avid metastatic disease (23,24). In fact, concerns over the frequency and severity of bone marrow and pulmonary toxicity associated with repeated high-dose RAI treatments were expressed in the 1950s and 1960s, which led to the development of dosimetry models to calculate the maximal tolerable activity that can be safely administered without causing excessive radiation exposure to the lungs or bone marrow (25). The value of this approach was recently discussed in a retrospective study demonstrating superior clinical outcomes when RAI therapy was based on whole-body dosimetry rather than empirical RAI dosing (13,14).

The inability to precisely define an optimal treatment activity of RAI means that clinicians cannot tailor treatment recommendations to individual patients. Therefore, some patients are treated with suboptimal doses and do not receive the complete potential therapeutic benefit of RAI. Conversely, some patients receive repeated RAI doses that are ineffective, which unnecessarily expose them to potential side effects. Salivary gland dysfunction, nasolacrimal obstruction, reproductive disturbances, conjunctivitis, and hematological abnormalities can be observed after RAI treatment (26). Although the majority of these side effects are minor and often transient, several studies have demonstrated that high cumulative administered activities of RAI given after traditional thyroid hormone withdrawal can be associated with leukopenia, thrombocytopenia, anemia, and even an increased risk of leukemia with cumulative doses >300-500 mCi (27,28).

## Conclusion

In conclusion, there is an unmet need for the development of accurate quantitative lesional and whole-body dosimetry that cannot be derived from diagnostic studies with the radioiodine isotopes currently used in practice. ^124^I dosimetry is the appropriate modality to achieve this, thus helping select patients likely to benefit from RAI therapy and sparing all others from unnecessary radiation exposure. Not only is ^124^I and PET imaging an exquisite methodology for research project such as ours, there is good reason to believe that ^124^I can be made into a practical clinical reagent to replace ^131^I for treatment dosimetry.
